# Review of Prevention for Pediatric and Adolescent Migraine

**DOI:** 10.15844/pedneurbriefs-30-1-4

**Published:** 2016-01

**Authors:** William Qubty, Amy A. Gelfand

**Affiliations:** 1 UCSF Pediatric Headache, University of California San Francisco, San Francisco, CA

**Keywords:** Migraine, Preventive, Pediatric Migraine, Prophylaxis

## Abstract

Authors from the Barrow Neurological Institute at Phoenix Children’s Hospital present a narrative overview of preventive treatment for pediatric and adolescent migraine.

Authors from the Barrow Neurological Institute at Phoenix Children’s Hospital present a narrative overview of preventive treatment for pediatric and adolescent migraine. Tricyclic antidepressants (TCAs) were thought to have the most evidence for pediatric migraine prevention, particularly amitriptyline and to a lesser extent nortriptyline. Serotonin selective reuptake inhibitors (SSRIs) have conflicting evidence for use in pediatric migraine. Selective serotonin and norepinephrine reuptake inhibitors (SSNRIs) including venlafaxine and duloxetine were thought to have more evidence for pediatric use. Antihypertensives including beta blockers, calcium channel blockers and angiotensin receptor blockers were discussed. The one with the most evidence in pediatric migraine was thought to be flunarizine, although it is not easily available in the U.S.. Propranolol, metoprolol, and candesartan may also be effective, however it is noted that the evidence is greater in adult studies. The anticonvulsant medications with the most support for use include topiramate and valproic acid. The antihistamine cyproheptadine was also recommended for preventive use. Finally, the authors review the evidence for botulinum toxin A injections. The authors admit the difficulty in drawing firm conclusions on its use in pediatrics based on the small, limited studies performed. They emphasize the need for effective migraine preventive measures in reducing headache-related disability and improving patients’ quality of life. [1]

COMMENTARY. Pediatric migraine is a common and potentially debilitating disorder. Migraine preventive treatment is commonly prescribed for young people affected by migraine at least 4 days per month or those who have experienced a significant impact on quality of life. This review proposes that preventive treatment can be considered successful when the patient has fewer than 4 migraine attacks per month which also subside with abortive therapy. In practical use, this definition of treatment success may be a bit stringent. A migraineur with 24 days of headache per month may be very satisfied if they are able to reduce this to 8 headache days per month. Alternatively, patients may also be satisfied with treatment if they are able to perform more routine daily activities even without a reduction in headache days. Data on what pediatric migraineurs, and their families, desire from preventive migraine treatment is a current gap in the literature.

This review has nicely summarized many of the typical migraine preventives used, common side effects, pertinent studies, and provides a concise list with starting dosage and dose range. To date there is no clinically available medication that was developed for the purpose of migraine prevention. However, there are promising recently completed phase IIb trials involving calcitonin gene related peptide (CGRP) antibodies for migraine prevention in adults [2] and hopefully these agents will ultimately be found to benefit children and adolescents as well.

Nutraceuticals including riboflavin, coenzyme Q10 and magnesium were not reviewed. Although the current evidence is limited, neutraceuticals may be an appropriate option for some patients. There is also increasing evidence in the adult literature for use of memantine, an NMDA antagonist [3]. Memantine is typically very well tolerated in our experience and has no monitoring requirements. Lastly, melatonin has been shown to be useful in adult migraine [4], and there is some evidence for its use in pediatrics [5]. Melatonin is typically well tolerated in children and adolescents and has minimal drug interactions [4].
